# Behind the curtain of the editorial process: how editors decide!

**DOI:** 10.1093/icvts/ivad022

**Published:** 2023-03-01

**Authors:** Peyman Sardari Nia, Ash Merrifield, Matthias Siepe

**Affiliations:** Department of Cardiothoracic Surgery, Maastricht University Medical Center, Maastricht, Netherlands; European Association for Cardio-Thoracic (EACTS) Surgery House, Windsor, UK; European Association for Cardio-Thoracic (EACTS) Surgery House, Windsor, UK; European Association for Cardio-Thoracic (EACTS) Surgery House, Windsor, UK; Department of Cardiac Surgery, Inselspital, Bern, Switzerland

**Keywords:** Peer-review, Editorial process

When you press the button to submit a manuscript, you are initially relieved, foregoing months or years of hard work. You depart for a moment from the grief of having been forced to confine your work into 5000 words. But after a while, there comes the anxiety. It is like sending your child to day care and wondering whether those taking care of your loved one do this with care. You wonder what happens in the abyss of the editorial process. You have some emotional attachment to it, as an artist has with her or his creation! However, what you do not realize is that in science, the most important part of any work is scientific scrutiny and improvement (= peer review).

Peer review is meant to improve your work, take out the emotion, de-commercialize it and provide the readers with your findings in the context of what has been done so far and what needs to be done.

The first thing you should know is that nobody has the authority to decide to reject, revise or accept any submission on their own. Although the Editors-In-Chiefs (EiCs) are the only ones who make the final decision, this decision is merely a confirmation of the ‘correctness’ of the process rather than a licence to decide anything one wants.

When a manuscript is submitted, it is checked by the editorial office and if the authors have followed the journal’s guidelines, it will officially enter the review process. It will first land on the desk of the appropriate associate editor (AE), with expertise on the subject. The AE can recommend rejecting the paper outright or decide to invite external referees. In the case of outright rejection, the manuscript will go to the EiC who—based on the arguments of AE—will decide on the outright reject. This is used when we judge that, even after peer review, the manuscript is not suitable nor has a high priority for publication.

All other submissions will be sent to referees that will be given 2 weeks (for original submissions, 1 week for revisions) to send their comments based on which the AE will recommend rejecting, revising or accepting.

Peer review is not perfect and there is without doubt some intertwining of ‘opinions’, ‘assumptions’ and ‘evidence’ in this process, and in what the authors have presented. Therefore, we are changing our editorial process to minimize the subjectivity in our peer review and increase transparency for everyone involved.

Our mission as the EiCs of *ICVTS* and *EJCTS* in the coming years will be to improve our peer review process by providing more uniformity, standardization and transparency to the authors. Therefore, we have changed our editorial processes as follows:

The reviewer’s scoresheet will be amended. The reviewers and editors will be asked to answer three main questions at the heart of the editorial decision-making:

1. Is it new?
2. Is it true?
3. Does anybody care?

The reviewers and editors need to select one response for each question from:

Yes

No

If No is selected, the reviewer/editor must explain why in maximum 380 characters.

The first question relates to the originality and novelty that is important in scientific publishing. It should be clear whether a paper provides novel insight or data of importance, and this should be done in a uniform manner for all our publications. The third question relates to whether the data provided would be of interest to our readership. By having clarity in how we judge the submission and restrict the answering of these questions to clear-cut answers we hope to have more standardization across the line.

The second question is the most important question. Nothing is ‘true’ or should be the ‘truth’ of what we publish eventually. Scientific knowledge should be surpassed by time. But what we mean as editors ‘Is it true?’ is whether the results are timeless based on the methods (confined to the Zeitgeist). This relates to the methods and statistics chosen that produce the findings and conclusions.

The nightmare of any respectable journal is to publish a manuscript and subsequently retract it. Here, we as editors rely on the judgement of referees and co-editors and on our own knowledge and experience to make decisions about the validity and robustness of data. We try our best to filter the bias out by having a process in which different people judge a submission.

We are forgoing the cover letter and instead will ask authors to complete a section in the editorial manager system as the editor’s appetizer. This is a structured section composed of three parts with a maximum of 200 words (excluding the references) as follows:

- Evidence before this study: here, the authors should highlight previous research published within the journal on this topic (cite at least two relevant references from recent publications in *ICVTS* or *EJCTS*).
- How your article adds to the topic.
- Implications of all the available evidence.

The editor’s appetizer invites and involves authors in the editorial process by providing standardized information that is needed to inform our editorial decisions and answer the question at the core of our decision-making process.

The next change is the standardization of the graphical abstract. We will ask authors to provide a uniform graphical abstract composed of two parts:

1. Summary of the core findings: if possible we recommend using Population, Intervention, Comparison, and Outcome structure with a maximum of 380 characters including the spaces. Here, we envision that authors explain in words the main summary of the manuscript’s methods and core findings.
2. Graphical representation of the core findings with a focus on numbers/statistics. Here, the focus is to present the main results in numbers or graphs.

We hope that these changes will provide greater clarity, transparency and objectivity to our editorial process. We acknowledge that peer review is never going to be perfect; however, we strive to improve as much as we can.

Finally, some editorial tips and tricks are as follows:

- Please consider that, in scientific publishing, it is all about having impact rather impact factor. Therefore, you need to submit your work to the right journal with the right readership that will read and cite your work.
- Nobody expects perfect written English. However, typos throughout the manuscript, especially in the title and abstract, do not reflect well on your work! We strongly recommend you ask that a native speaker checks your work before submission.
- Science is about numbers and how you got to those numbers. Writing an abstract without clearly specifying which kind of study you have conducted, in which population with which methods and what results you got in numbers, just will not work well. As editors (and interested readers), we do not want to dig deep into the manuscript trying to find whether the study was prospective or retrospective, what the sample size was, what the numbers in each group were, etc.
- Introduction of an original contribution should be no more than 3 paragraphs or even less: please be aware that you are not writing a review paper! Long introduction: here, you will lose the interest of your readers. End the introduction with a clear question or hypothesis.
- Methods: this is the most important part of the manuscript. Here, we look for robustness, validity and timelessness. Timelessness is about publishing something that in retrospect even if the results would not hold, still the methods used for the cohort to generate the results should hold.
- Results section should be presented in a raw manner without interpretation and without repetition. Often we see that numbers in abstracts, tables and results sections are different or do not add up correctly!
- Discussion: first explain your findings and then compare your finding with published data, then the implications, future perspectives, and finally, the limitations. The reviewers are experts, and they might see the limitations immediately: omitting these limitations doesn't reflect well on your scientific integrity.
- The conclusions should match the results and should not reflect an unjustified generalization.
- Please do not use language that reflects your emotional attachment to your work like extremely significant, very critical finding, the only group in the world with these results, etc.
- When you are invited to resubmit your revisions, please consider that as editors we want to see a genuine effort of addressing the concerns of the reviewers and the improvements that we have envisioned. Do not simply argue against the critique; most referee remarks should lead to changes in the manuscript. Without it, there is a slim chance of acceptance.

## Notes

This article has been co-published in European Journal of Cardio-Thoracic Surgery and Interdisciplinary CardioVascular and Thoracic Surgery.

